# Development of Seed Butter Made with Pumpkin, Sesame, and Sunflower Seeds and the Influence of Natural Antimicrobials and Stabilizers on Its Shelf Life

**DOI:** 10.1155/2021/6630636

**Published:** 2021-03-22

**Authors:** Yung-Hsin Chien, Melvin A. Pascall

**Affiliations:** Department of Food Science and Technology, The Ohio State University, USA

## Abstract

This study investigated the antimicrobial efficacies of grape seed extract (GSE) and cinnamaldehyde (CIN) against *Salmonella enterica* and *Listeria innocua* and the influence of hydrogenated rapeseed oil (HRO) and palm kernel oil (PKO) on the texture and oil separation in pumpkin/sesame/sunflower seed butter. The results showed that the 10 and 15% GSE significantly reduced both *S. enterica* and *L. innocua*. Cinnamaldehyde was effective against *S. enterica* but did not significantly reduce *L. innocua*. Hydrogenated rapeseed oil at 2 and 3% concentrations prevented hardening of the seed butter and thus facilitated its spreadability. The 3% HRO-stabilized seed butter had less oil separation and a better texture than the control. Although PKO influenced the hardness of the butter after 35 days, its effect was not as pronounced as that of HRO. The HRO was also more effective in reducing the adhesiveness and thus the stickiness of the seed butter when compared with the PKO. Both HRO and PKO did not influence cohesiveness and adhesiveness changes to the butter after 7 days, although the HRO samples showed a lower level of cohesiveness when initially added to the samples.

## 1. Introduction

Seed butter is a spreadable product made by milling seeds into a paste. Pumpkin, sesame and sunflower seeds, and soybean are common ingredients used to make seed butter. Similar products can be made from various nuts, including almond, cashew, hazelnut, macadamia, peanut, pecan, pistachio, and walnut. Seed butter is a good source of protein, in addition to other essential nutrients [1]. Seed butter could be consumed in a variety of ways as a spread on bakery products, as a dip for vegetables, and as a filling in candies and in drinks and desserts such as ice creams and smoothies [2].

The shelf life of seed butter could be influenced by factors such as microbial contamination, lipid oxidation, and texture changes. Microbial contamination could be associated with bacteria and/or fungi and could cause spoilage or become a safety issue if pathogens are involved. Since seeds have a high lipid content, the oil could be susceptible to oxidation if the product is stored in conditions that favor rancidity, especially in the absence of appropriate antioxidants. Depending on the particle size of the milled seeds, granule segregation could take place, and larger and heavier particles could sink to the bottom of the container while smaller and lighter ones, plus the oil, could settle in the upper layers [3]. This has the potential to cause the lower solid layers to harden and increase the difficulty in removing the product from the container and spreading it onto items such as bakery products. If the oil that rises to the top of the seed butter is exposed to air in the headspace of the container, it could become oxidized, induce rancidity, and shorten the product's shelf life.

Within recent times, several reports of outbreaks associated with peanut butter have occurred within the United States. The first highly publicized outbreak caused by *Salmonella* in peanut butter occurred in 2006, and at least 628 consumers from 47 states were infected [4]. One year later, another outbreak occurred, and 529 consumers from 43 states were infected by *Salmonella* in peanut butter and in peanut butter cookies. In addition to these, widespread outbreaks occurred in 2012 (when 41 people from 20 states were infected) and in 2014 (when 6 more consumers from 5 states were infected) [5]. Not only was peanut butter the affected substrate, sesame paste was also contaminated by *Salmonella* in 2013 and 16 consumers from 5 states were infected [6]. Seed butter can be contaminated as a result of poor sanitary practices, inappropriate equipment design, and improper ingredient control. Although vegetative microbial pathogens poorly survive in low-water activity foods such as seed butter, the literature reports that some microorganisms are able to tolerate these conditions for given periods of time and could be sources of food safety problems [7].

To reduce the incidence of spoilage and a shortening of the shelf life of seed butter, several hurdles should be adapted. Although good manufacturing practices are known to minimize the potential for microbial contamination, the use of antimicrobial agents in the product could be an added bonus in the event of accidental microbial inoculation. In the case of rancidity, it could be minimized using appropriate antioxidants, especially since many consumers usually store seed butter at room temperature. To reduce the potential for oil separation in seed butter, an appropriate stabilizer or emulsifying agent should be added to the product.

Peanut butter is the most widely consumed seed butter. However, peanuts and other tree nuts are known allergens and cannot be consumed by a fairly large percentage of consumers. Thus, an alternative to peanut butter, but with similar taste, texture, and nutritive value, is an advantage. The objectives of this study were the following: (1) to develop an alternative to peanut butter made from seeds that are considered nonallergenic in the United States, (2) to test the antimicrobial efficacies of two ingredients in the seed butter, and (3) to select a vegetable oil for use as an appropriated homogenizer for improved texture of the seed butter.

## 2. Materials and Methods

### 2.1. Materials

The *Salmonella enterica* (ATCC 53647) and *Listeria innocua* (ATCC 33090) used in this study were purchased from the American Type Culture Collection (Manassas, VA). Tryptic soy broth (TSB) and tryptic soy agar purchased from Difco (Sparks, MD) were used to grow the bacteria. Grape seed powder (extract) marketed as Leucoselect® and containing ≥95.0% to ≤105.0% proanthocyanidins, as determined by Gel permeation chromatography, and ≥13.0% to ≤19.0% catechin and epicatechin, as determined by high pressure liquid chromatography, obtained from Indena S.p.A. (Milan, Italy) was used in this study. The cinnamaldehyde (CIN) was obtained from Parchem Chemicals (New Rochelle, NY).

### 2.2. Seed Butter Production

The seed butter was prepared using the formulation shown in Table 1. The ingredients were grounded in two stages. In the first stage, they were blended and grounded at 1500 rpm and 50°C in a Stephen UMC 5 electronic mixer (Stephen Food Service Equipment GmbH, Halen, Germany) for 2 minutes. In the second stage, the ingredients were grounded at 3000 rpm and 50°C for 16 minutes. The seed butter was then cooled to 23 ± 1°C before storage and various analyses.

### 2.3. Culture Preparation

The stock cultures of *Salmonella enterica* and *Listeria innocua* were prepared by transferring a loopful of each organism into 30 mL of TSB followed by incubation at 37°C for 24 hours. Each broth was centrifuged (Kendro Laboratory Products, Sorvall RC 5C Plus, Newtown, CT, USA) at 6,000 rpm and 4°C for 10 minutes. The supernatant was decanted, and the suspension was resuspended in 30 mL 0.85% sterile saline (pH 6.5). Prior to the inoculation of the seed butter samples, the microbial solutions were analyzed to determine the bacterial populations (CFU/mL) by being serially diluted (1 : 10) in 0.85% sterile saline and pour plated (1 mL in duplicate) with tryptic soy agar (TSA). All the plates were incubated at 37°C for 48 hours and then counted.

### 2.4. Sample Preparation for Microbial Testing

For each analysis, 20 g aliquots of seed butter with 0, 5, 10, and 15% GSE or 0, 0.1, 1, and 1.5% CIN were placed in sterile stomacher bags (240 mL) (Fisher Scientific, Pittsburgh, PA) and inoculated with 200 *μ*l of *S. enterica* (10^7^–10^8^ CFU/mL) or *L. innocua* (10^7^–10^8^ CFU/mL), respectively. The samples were then homogenized in the stomacher for 2 minutes at 23 ± 1°C. The sterile stomacher bags with the preinoculated seed butter were sealed and stored at 25°C for up to 9 days.

### 2.5. Microbial Analysis

The inoculated seed butter samples were stored for 1, 3, 5, 7, and 9 days at 25°C then analyzed for populations of *S. enterica* and *L. innocua*. To each stomacher bag, a 50 mL aliquot of 0.85% sterile saline was added, and the mixture was homogenized in the stomacher for 2 minutes. Each sample was serially diluted (1 : 10) in 0.85% sterile saline then pour plated with TSA. All samples were incubated at 37°C for 48 hours before inspection for presumptive *S. enterica* and *L. innocua* colonies.

### 2.6. Texture Profile Analysis

This experiment was done to determine the effects of HRO and PKO on the texture of the seed butter samples during storage at different temperatures. For the texture profile analysis, a Texture Analyzer TA-XT2 (Texture Technologies Corp and by Stable Micro Systems, Ltd., Hamilton, MA, USA) was used to measure the hardness, cohesiveness, and adhesiveness of the seed butter samples. The method was modified from the one reported by Radočaj et al. [8]. The Texture Analyzer was fitted with a 5 kg load cell. The resulting force-time curves were generated to calculate the hardness, adhesiveness, and cohesiveness of the seed butter samples during storage at 25°C. All samples were tested within 24 hours of production.

A cone-shaped acrylic probe (40^0^) was used for all measurements, and each sample (150 g) was tested in the sample holding container (cylinder shape: 45 mm depth and 36 mm diameter) without stirring. The pretest speed was 2 mm/s, and the test speed was 1 mm/s. The target depth was 42 mm. The trigger load was 4.0 g, and the data rate was 100 points/s. After each test, the probe automatically returned to its original position for testing the next sample. The instrumental texture attributes tested were as follows: (1) hardness—the peak force required by the cone-shaped probe to compress the sample, (2) adhesiveness—the work required to pull the cone-shaped probe away from the surface of the sample, and (3) cohesiveness—the strength of internal bonds in the seed butter matrix.

### 2.7. Oil Separation

This experiment was conducted to determine the effects of HRO and PKO on inhibiting oil separation in the seed butter. The method used was modified from the one reported by Ereifej et al. [9]. For each test, 50 g of the seed butter with 0, 1, 2, and 3% concentrations of HRO or PKO was loaded in a plastic cup (100 mL capacity) and covered with perforated aluminum foil (1.5625 hole/cm^2^). The cup was inverted and placed in a Petri dish which had 5 sheets of filter paper (Schleicher & Schuell No. 589). This was designed to absorb the oil that separated from the seed butter. The weights of the Petri dishes with the absorbed oil on the filter paper were measured on 0, 1, 3, 5, 7, 14, 21, 28, 36, and 42 days of storage in order to determine the oil separation characteristics of the samples. This oil separation test was evaluated at 25°C and determined from the following equation:

(1) $$\begin{array}{c} \text{Oil separation percentage}=\left(\frac{B-A}{\text{Original weight of oil}}\right)\times 100, \end{array}$$

where percent oil separation was calculated by taking away the initial weight (g) of the filter paper and Petri dish (*A*), from the weight (g) of the filter paper, separated oil, and Petri dish (*B*). This value was divided by the original weight of the seed butter at the start of the test. To obtain the percent oil separation, this figure was multiplied by 100.

### 2.8. Statistical Analysis

This was conducted using the JMP 10 statistical software (SAS Institute Inc., Cary, NC, USA) for one-way Analysis of Variance (ANOVA) and Tukey-Kramer testing to determine the inhibitory effects of different levels of GSE and cinnamaldehyde against *S. enterica* and *L. innocua* and the efficacies of different concentrations of HRO and PKO on the physical and textural properties of the seed butter. The confidence interval was set at 95%, and the data was collected in triplicate. The entire experiment was repeated three times.

## 3. Results and Discussion

### 3.1. Antimicrobial Activity of Grape Seed Extract in the Seed Butter

**Figure 1:**
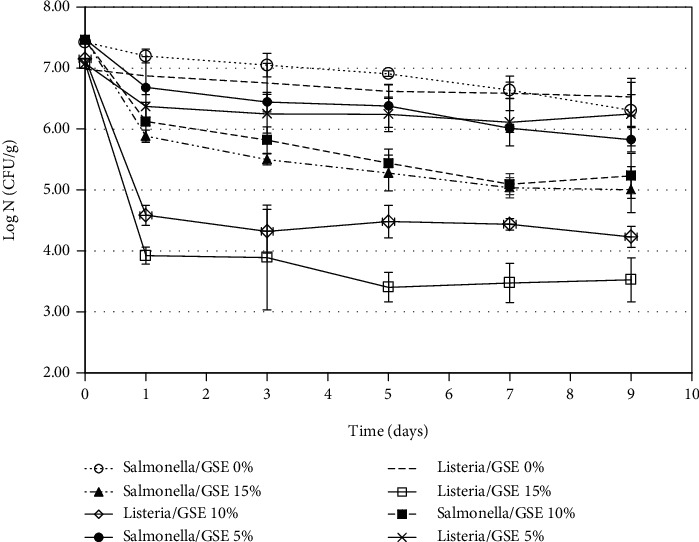
Inhibitory effect of grape seed extract against *S. enterica* and *L. innocua* at 25°C.

The antimicrobial activities of the GSE incorporated into the seed butter are shown in Figure 1. Compared with the control, the *S. enterica* numbers significantly (*p* < 0.05) decreased after one day of storage in 10 and 15% GSE seed butters, but there was no significant (*p* > 0.05) difference in the *S. enterica* loads between the 10 and 15% GSE seed butters. Although the 5% GSE also lowered the number of *S. enterica*, it was less effective than the higher concentrations and was still significantly (*p* < 0.05) more effective when compared with the control.

For the antimicrobial activity of GSE against *L. innocua*, after one day of incubation, the reductions were significantly (*p* < 0.05) higher in the 10 and 15% GSE seed butters when compared with the control and the 5% GSE seed butter. *L. innocua* survival in the 5% GSE seed butter was not significantly (*p* > 0.05) different from that of the control. There were significant (*p* < 0.05) differences in *L. innocua* reductions in the 10% when compared with the 15% GSE seed butter after 5 days of storage.

Friedman et al. [10] studied and reported on the antimicrobial properties of GSE against *S. enterica*. Additionally, Rhodes et al. [11] investigated the antimicrobial potential of GSE against *L. monocytogenes* and reported a 6–7 log CFU/mL microbial reduction within 10 minutes at 20°C. The findings of Over et al. [12] and Rhodes et al. [11] showed that the antimicrobial effect of GSE against the microorganism occurred in nutrient media. Our study took a more realistic approach and tested the antimicrobial abilities of GSE against *S. enterica* and *L. innocua* in a specific and more complicated food system, seed butter, which contained fatty acids, proteins, carbohydrates, and other minerals.

Grape seed (*Vitis vinifera*) extract is a by-product from grape juice and wine processing. Tseng and Zhao [13] reported that GSE is rich in polyphenolic compounds with catechins being the major active components. Weber et al. [14] reported that these catechins are capable of combining with gallic acid to form gallate esters but could also exist in monomeric phenolic compounds such as epicatechin and epicatechin-3-O-gallate, or in dimeric, trimeric, and tetrameric procyanidin forms. Proanthocyanidins are also found in GSE and are dimeric, trimeric and tetrameric forms of procyanidin. These polyphenols are known to deactivate bacteria by acting on the outer cellular and/or cytoplasmic membranes. They cause damage to the bacterial as a result of alterations to the osmotic pressure of the cytoplasm and a resultant leakage of cell constituents [15]. Amankwaah et al. [16] also reported on the antimicrobial properties of GSE and even showed that it had antiviral properties when in broth and when incorporated into edible starch-based films.

### 3.2. Antimicrobial Activity of Cinnamaldehyde in the Seed Butter

**Figure 2:**
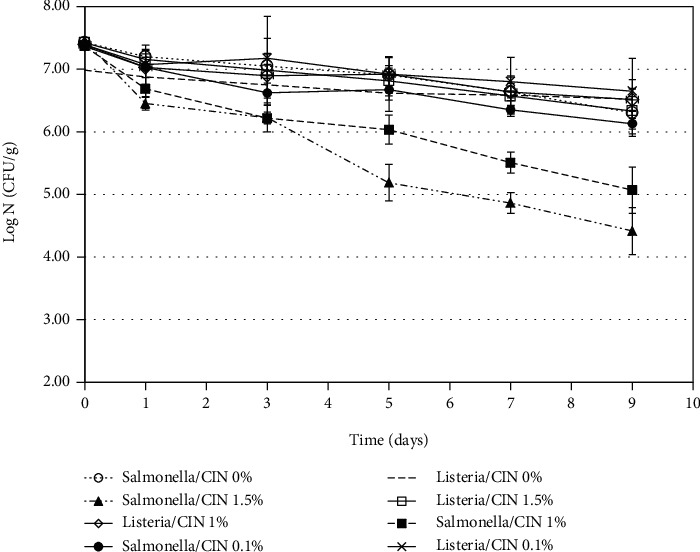
Inhibitory effect of cinnamaldehyde against *S. enterica* and *L. innocua* at 25°C.

The antimicrobial efficacy of CIN in the seed butter was investigated against *S. enterica* and *L. innocua*. Seed butter without the added antimicrobial agents was used as the control.  Figure 2 summarizes the results obtained and shows that there was no significant (*p* > 0.05) difference in the *S. enterica* counts between each treatment until the fifth day of storage. Subsequently, reduction in *S. enterica* in the 1.5% CIN seed butter (2.26 log CFU/g) was significantly (*p* < 0.05) higher than the reductions in the other seed butter samples. After 7 days of storage, the 1 and 1.5% CIN seed butters significantly (*p* < 0.05) reduced *S. enterica* counts by 1.86 and 2.59 log CFU/g, when compared with the control and the 0.1% CIN seed butter. There were significant (*p* < 0.05) reductions in *S. enterica* between the 1.0 and 1.5% CIN seed butters. On the other hand, Figure 2 also shows that seed butters with 0.1, 1.0, and 1.5% CIN did not significantly (*p* > 0.05) reduce *L. innocua* after 9 days of storage at 25°C when compared with the control.

The antimicrobial properties of CIN have been reported by Ravishankar et al. [17] who stated that a 0.1% concentration in a phosphate-buffered saline (PBS) solution against *S. enterica* ser. Typhimurium, *S. enterica* ser. Enteritidis, and *S. newport* produced 5.0 log CFU/mL reductions in 1 hour at 37°C. However, further testing showed that *L. innocua* was more resistant to the antimicrobial effects of CIN. Similar results were also mentioned in a report by Ravishankar et al. [18]. *L. monocytogenes* also showed resistance to CIN and showed a nonsignificant (*p* > 0.05) reduction when compared with the control in a study from de Oliveira et al. [19]. The findings by these researchers are similar to what we found when we investigated the effectiveness of CIN in the seed butters.

The literature reports that the main compounds found in cinnamon are cinnamic aldehyde (cinnamaldehyde), coumarin, cinnamic alcohol, *α*-copaene, and benzenepropanal. Of these compounds, cinnamic aldehyde is the major compound, and it acts to inhibit bacterial growth by interfering with the activities of some of its enzymes [20, 21]. Cinnamaldehyde is a pale-yellow viscous liquid and the major compound that gives cinnamon its flavor and odor. It occurs naturally in the bark of the cinnamon plant (genus *Cinnamomum*).

### 3.3. Texture Profile Analysis

**Figure 3:**
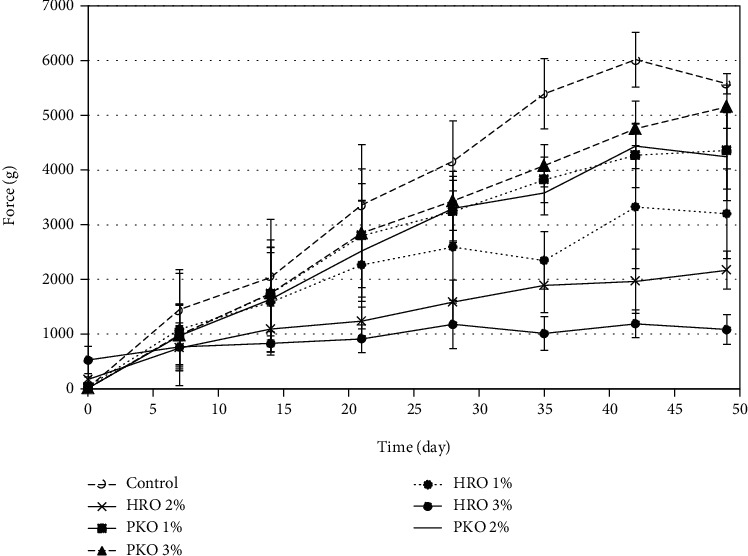
The efficacy of HRO and PKO on the hardness of the seed butter (1000 g = 9.81 Newtons).

The impact of HRO on the hardness of the seed butter samples is presented in Figures 3, and it shows that seed butter containing 3% HRO was significantly (*p* < 0.05) lower in hardness than all the other samples after the 28 days of storage. No significant difference (*p* > 0.05) existed between the hardness of the samples with the 1 and 2% HRO stabilizer. However, after 28 days of storage, the 1 and 2% HRO samples were significantly lower in hardness (*p* < 0.05) than the control. After 35 days of storage, the seed butter samples stabilized with PKO were significantly (*p* < 0.05) lower in hardness than the control (Figure 3). However, there was no significant (*p* > 0.05) difference between the different levels of PKO on the hardness of the seed butter. The peak hardness of the control occurred on day 42, but it dropped by 439.2 g/force on day 49.

**Figure 4:**
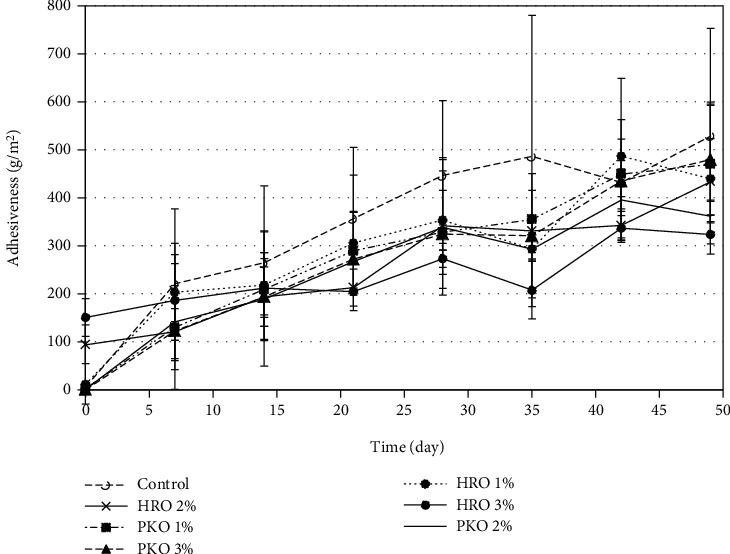
The efficacies of HRO and PKO on the adhesiveness of the seed butter.

Figure 4 shows the effects of HRO on the adhesiveness of the seed butter. The figure shows that samples without added HRO had the highest level of adhesiveness. The results also show that the 3% HRO samples had the lowest level of adhesiveness. However, the statistical analyses show that the differences in the adhesiveness between all samples were not significant (*p* > 0.05). The results for the effect of PKO on the adhesiveness of the seed butter samples were similar to those obtained for the HRO. In general, the results show that the control had the highest level of adhesiveness but still the differences were not significant (*p* > 0.05) from those of the 1, 2, and 3% PKO-treated samples.

**Figure 5:**
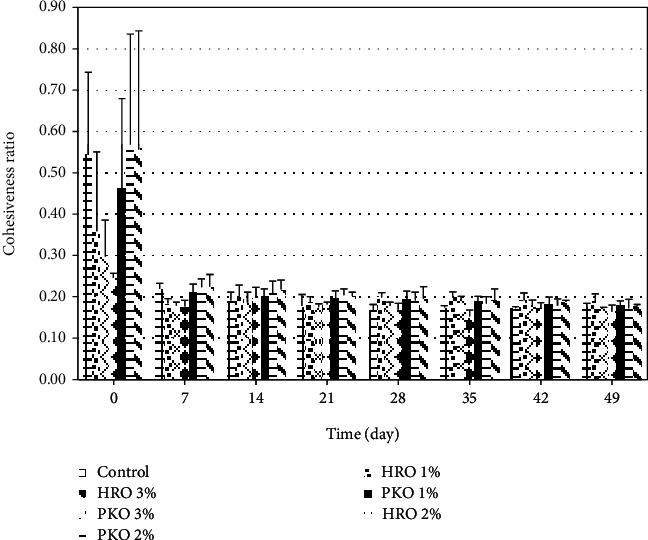
The efficacies of HRO and PKO on the cohesiveness of the seed butter.

The results of the effects of HRO and PKO on the cohesiveness of the seed butter samples are shown in Figure 5. For the effects of the HRO, the figure shows that the 3% concentration had a significant (*p* < 0.05) effect on the cohesiveness when initially added to the seed butter samples and up to day 7 of storage. During that time, the 3% concentration was significantly different from the control but not significantly (*p* > 0.05) different from the 1 or the 2% HRO samples. For the remainder of the storage period (between days 14 to 49), there was no significant (*p* > 0.05) difference in the effect of HRO on the cohesiveness. On day 7 of storage, although there was no significance between the mean cohesiveness for the 1, 2, and 3% HRO concentrations, when considered together, these concentrations were significantly (*p* < 0.05) different from the control. For the samples treated with the PKO, initially there were no significant (*p* > 0.05) differences in the effect of concentration on the cohesiveness. The results for all concentrations were significantly (*p* < 0.05) different after day 7 of storage when compared with the results from day 0.

### 3.4. Oil Separation

**Figure 6:**
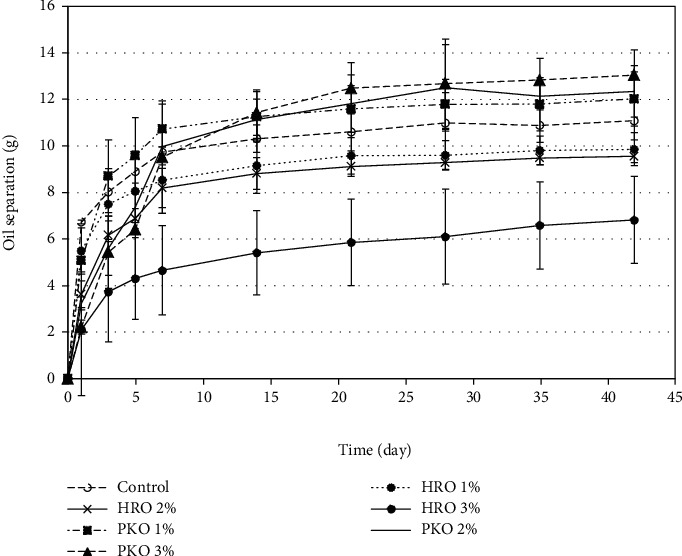
The efficacies of HRO and PKO on the oil separation in the seed butter.

When the HRO was used as the stabilizer, Figure 6 shows that the 3% concentration had a significant effect (*p* < 0.05) on reducing the oil separation in the seed butter. Figure 6 also shows the effects of PKO on preventing the oil separation. For all concentrations of the PKO in the oil, the figure shows that the oil separation stabilized after 7 days of storage. However, the figure shows that PKO had no significant effects (*p* > 0.05) on the level of oil separation. The figure also shows that the addition of PKO promoted more oil separation since the control had a lower level when compared with the other samples.

The hardness of seed butter samples could increase during storage if the oil in the product migrates toward the surface and gravitational settling of the solid particles occurs at the bottom of the container [22]. The results of our study showed that the 3% HRO maintained the consistency of the samples at an almost constant level at 25°C for at least 42 days. The results also showed that the stabilizing effect of HRO increased with increasing concentrations. On the other hand, PKO did not show the same stabilizing effects. These results are in agreement with a similar study reported by Shakerardekani et al. [23], who showed that palm oil in pistachio spread did not significantly (*p* > 0.05) contribute to its consistency. In studies done by Smith et al. [24], they showed that HRO provided a stable crystallization network structure when in seed butter matrix and this helped in maintaining a consistent hardness profile and thus prevented the oil migration. Concerning PKO, Berger [25] reported that it only contained 17% solid fat content at 25°C, and this characteristic may be a partial reason why it was ineffective as a stabilizer in the seed butter in our study. Compared with rapeseed oil, which has 49% erucic acid, this 22-carbon monounsaturated acid forms an effective crystalline structure after hydrogenation [26], and this probably helped the HRO in our study to provide a better support structure for homogeneity of the seed butter particles.

The seed butter texture profile analysis (TPA) obtained for this study was done by compressing and decompressing the samples on a flat plate twice by a probe attached to a drive system. This simulated the chewing action of the teeth. From the force (on the *y*-axis) vs. time (on the *x*-axis), the hardness of a sample was the peak force (that simulated the first bite by an individual), and it was the resistance of the sample to the applied force. As the jaw of the individual opens prior to the second bite, the energy required to overcome the stickiness of the sample on the teeth and the palate is referred to as the adhesiveness of the sample. In testing the textural properties of peanut butter, Yadav [27] reported adhesiveness as the negative area of the force vs. time curve. During the TPA, this would be the work required to pull the compression probe from the sample [28]. In the second bite by the individual, it would be obvious that less force would be required to crush the sample because of the downward force of the first bite. The resistance of the sample to the second bite would thus be influenced more by the molecular structure of the sample. The ratio of the area under the second bite curve to that of the area under the first bit curve is defined as the cohesiveness of the sample [29]. It is thus the structural integrity of the sample as it opposes the compressive forces of the probe. This simulates the energy needed by an individual to break down the food prior to swallowing. This also has implications for the spreadability of the seed butter. If the stabilizing effect is too high, it would cause the product to be too hard and this would increase the difficulty of removing it from a container and the ease of spreading it on a substrate [23].

Hardness and consistency have a direct correlation to the spreadability of seed butters normally used by consumers [27]. If the hardness is too high, the result would be a product that is difficult to spread on food items. At the same time, if hardness is too low, the product would be too soft and would flow easily. Since seed butters made from peanut, cashew, almond, sunflower, sesame, and other types of seeds are most often used as spreads on bakery products, it is essential that its consistency for such applications be convenient for use by consumers. If a seed butter has a relatively low hardness rating, its use for making liquid beverages would be more applicable.

In relating the results of the TPA to the seed butter samples tested in this study, if the adhesiveness was too high, this would be unacceptable to consumers since the product would tend to stick to the teeth during consumption. Also, a high adhesive value would mean that the seed butter would tend to stick too tightly to a tableware item and become a source of inconvenience. The results of our study showed that the seed butter stabilized with 3% HRO had the lowest adhesiveness during storage. This result agreed with the data reported by Aryana et al. [3] who showed that hydrogenated rapeseed and cotton seed oils effectively prevented an increase in adhesiveness in peanut butter up to 23 weeks of storage. Since seed butter could be described as a semisolid food, its gumminess would be defined as the product of its hardness multiplied by its cohesiveness [29]. To determine the optimum hardness, adhesiveness, and cohesiveness of the seed butter in our study, it would be best to correlate the instrumental data with sensory studies. This will form the basis of additional studies. However, the use of instrumental analyses is essential as a precursor to sensory studies as reported by several searchers [26, 30, 31].

## 4. Conclusion

The results of this study showed that as an antimicrobial additive, GSE effectively reduced the load of *S. enterica* and *L. innocua* in the seed butter at 25°C. Although CIN effectively inhibited *S. enterica* at 25°C, its effect against *L. innocua* was not as effective. The addition of HRO to the seed butter samples prevented significant migration of the oil to the surface up to 49 days of storage. Its effectiveness on the adhesiveness and cohesiveness of the samples was not as extensive. The addition of PKO did not show the same level of effectiveness on the physical properties of the seed butter samples when compared with the HRO.

## Figures and Tables

**Table 1:** Ingredients and formulation.

Ingredients	% in seed butter
Pumpkin seed^a^	32.87
Sunflower seed^a^	26.10
Sesame seed paste (Tahini)^b^	19.14
Honey^c^	19.14
Salt (sodium chloride)	2.75

^a^Pumpkin and sunflower seeds were purchased from King Nut Co. Inc. (Solon, OH). ^b^Sesame seed paste (Tahini) was purchased from International Golden Foods, Inc. (Bensenville, IL). ^c^Honey was purchased from Golden Food Service (Columbus, OH).

## Data Availability

The data used to support this study are available from the corresponding author upon request.
